# Traumatic Bilateral Asymmetric Hip Dislocation With a Rotated Pipkin Type II Femoral Head Fracture

**DOI:** 10.5435/JAAOSGlobal-D-25-00128

**Published:** 2026-02-05

**Authors:** Taif Al-Jafar, Abdulazeez Ismail, Ali Al-Hilli

**Affiliations:** From the University of Baghdad, College of Medicine, Baghdad, Iraq (Dr. Al-Jafar and Dr. Ismail), and the Department of Surgery, University of Baghdad, College of Medicine, Baghdad, Iraq (Dr. Al-Hilli).

## Abstract

Bilateral asymmetric hip dislocation is a rare injury with a complex mechanism, and treatment of delayed cases is difficult with unpredictable outcomes. We report a case of a 25-year-old man with bilateral asymmetric hip joint dislocation (right anterior, left posterior) and a Pipkin type II left femoral head fracture. His dislocations were reduced under sedation within 4 hours of the accident, but the surgical fixation of his fracture was delayed for 4 days. Hip dislocation should be reduced within 6 hours of injury. Postreduction CT in high-energy trauma is especially crucial for detecting associated fractures. If closed reduction fails, then open reduction should be conducted to prevent complications such as osteonecrosis, heterotopic ossification, and osteoarthritis.

Bilateral hip dislocation is a rare injury accounting for approximately 1% of hip^1^ dislocations and 0.01% to 0.02% of all joint dislocations.^2^ The major cause is usually high-energy trauma such as a motor vehicle accident or fall from height. The exact mechanism of injury is not fully apparent because of its complexity, the high velocity required, and the opposing forces needed to dislocate asymmetrically. Both hips might dislocate simultaneously, or the dislocations might be temporally separate instances. The notable force required for such an injury makes it logical to look for associated injuries, notably of the acetabulum or proximal femur. A study by Hougaard et al. found that 88% of hips reduced within 6 hours had excellent or good results, compared with only 42% when reduction was delayed beyond 6 hours.^3^ In this study, we present the rare case of a 25-year-old man with bilateral asymmetric hip dislocation and a Pipkin type II left femoral head fracture, where the dislocations were reduced within 4 hours but the fracture fixation was delayed for 4 days, with 2 years of follow-up documentation.

## Case Presentation

The patient is a 25-year-old man who was involved in a motor vehicle accident while driving carelessly using his cellphone. The patient struck a concrete block at approximately 40 miles per hour. He was not under the influence of drugs or alcohol. The patient was unrestrained by a seat belt and sustained multiple injuries, including fractures in the nasal, zygomatic, and mandibular bones; right comminuted talar head and neck fracture; and left femoral head fracture, as well as bilateral asymmetric hip joint dislocation.

**Figure 1:**
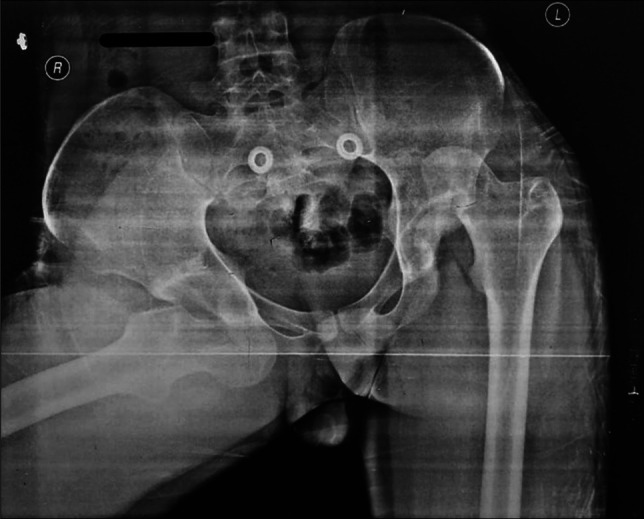
Pre-reduction pelvic radiograph, showing bilateral (right anterior and left posterior) dislocations of the hip.

**Figure 2:**
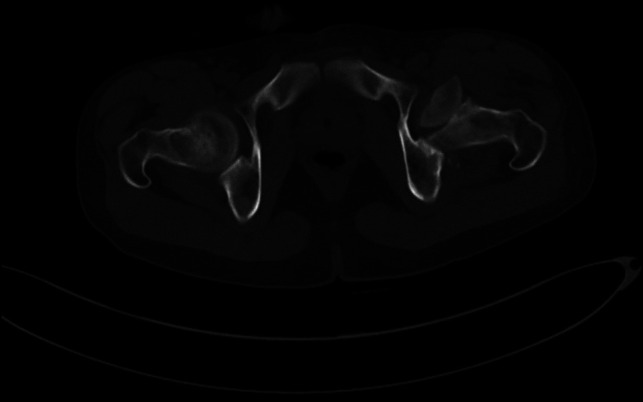
Postreduction preoperative CT scan showing the fracture and the rotated left femoral head fragment.

The patient was immediately transported to the nearest hospital where he was stabilized. The patient was lying on his right side, refusing to be adjusted because of pain, with his left hip slightly flexed, adducted, and internally rotated, indicating a posterior dislocation, whereas his right hip was flexed, abducted, and externally rotated, indicating an anterior dislocation.^4^ Radiographs confirmed the dislocations (Figure 1), which were promptly reduced under sedation, but no postreduction imaging was taken, which led to the fracture in the left femoral head being unrecognized. On the second day, the patient developed severe pain and swelling in his hip and he was transferred to another hospital, but the left hip that had a Pipkin type II femoral head fracture was not addressed there because of a lack of resources (Figure 2). On the third day, the patient was transferred to our specialized hospital for additional treatment. He was hemodynamically stable with a Glasgow Coma Scale score of 15.

Definitive surgical intervention was delayed until the 4th day after the accident. The dislocation of the left hip was initially reduced when he was transported to the nearest hospital after the accident, but the fractured piece of the femoral head remained rotated until it was relocated intraoperatively through a Kocher-Langenbeck approach, revealing multiple tears in the posterior hip capsule, as well as severe cartilage damage. The wound was debrided, and then, fixation of the femoral head fracture was performed using two Herbert screws. The hip was reduced, and the posterior capsule and short external rotators were repaired. The patient's other fractures were also addressed during the same operation. Postoperatively, the patient was given thromboprophylaxis with enoxaparin.

Owing to his mandibular fracture, his mouth was wired shut, necessitating enteral feeding through a syringe. He was restricted to a liquid diet for 6 weeks, primarily milk-based, leading to notable weight loss and malnutrition. Oral lactulose was required postoperatively because of severe constipation, culminating in an emergency department visit for fecal disimpaction.

The patient began standing with crutches 5 weeks after surgery and was fully weight bearing with an ankle orthosis after 8 months. However, he exhibited poor compliance with physical therapy, leading to functional limitations. He reported chronic right ankle pain with swelling, reduced dorsiflexion and plantarflexion, and Achilles tendon tightness. Imaging 3 months after the accident demonstrated signs of union in the left femoral head fracture. Six months after surgery, follow-up imaging showed no signs of osteonecrosis in the left hip.

His follow-up 19 months after the accident revealed a mild limp on the right side, with a balanced pelvis and normal spinal curvature. Lower limb reflexes and sensory function were intact, but he displayed persistent limitations in right ankle inversion, eversion, and plantarflexion.

**Figure 3:**
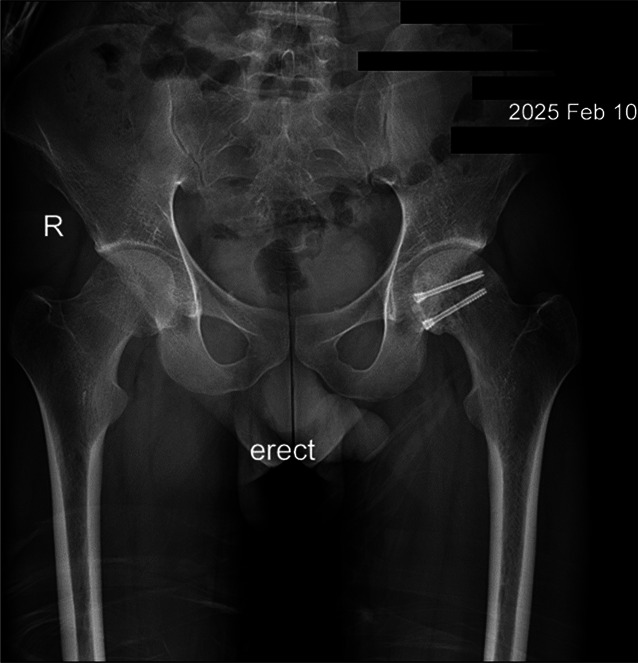
Two-year follow-up pelvic radiograph, showing the two Herbert screws used to fixate the left femoral head.

At the last follow-up, 23 months after the accident (Figure 3), he had full range of motion in both hips and knees, with no discomfort or pain during passive or active movement. The right ankle remained with painless limited range of motion in all directions due to fracture malunion, which led to difficulty running and squatting. Despite these deficits, he reported no notable psychological effect.

Overall, the patient has returned to work as an electrician but continues to experience activity-related discomfort, particularly in his right ankle and rarely in the left hip with extended periods of standing.

## Discussion

The hip joint is a congruent joint and one of the most stable joints in the body because of its strong ligaments, acetabular depth with the labrum, thick capsule, and strong surrounding muscles. This implies the necessity of a high-energy trauma to cause any fractures or dislocations in a healthy young adult. Hip dislocations are more common in young adult men.^2^ In this case, the patient, a 25-year-old man, suffered a head-on collision with a concrete block while unrestrained by a seat belt. This caused a dashboard injury, which is the main cause of the bilateral dislocation.^5^

The biomechanics that are required to produce this injury include adduction of the hip joint for posterior dislocation and abduction for anterior dislocation. This can only be achieved in a motor vehicle accident, and less commonly, a fall from a height. Owing to the high-energy nature of the offending trauma, it is important to look for other associated injuries, especially in the head, thorax, and abdomen. Our patient suffered a comminuted talar fracture and a steering wheel injury that led to zygomatic, maxillary, and mandibular fractures, but with no internal bleeding in any of the body cavities.

Clinical examination is the most important diagnostic tool for dislocations, which need to be confirmed by plain radiography. It is necessary to take both pre-reduction and postreduction radiographs to ensure that the dislocation was reduced properly. This must be done quickly to reduce the risk of the most common complications such as osteonecrosis, heterotopic calcification, and osteoarthritis.^6^ The Incidence of these complications increases the more the reduction is delayed.^7^ These are caused by damage to the blood supply of the femoral head.^8^ In our case, the fractured femoral head fragment is certainly deprived of blood supply, but early and accurate reduction and fixation of the fracture improve the chances of revascularization.

In our case, despite the fact that surgical intervention for the Pipkin type II left femoral head fracture was delayed for 4 days from the time of the accident, the patient suffered no permanent hip disability and recovered fully. We believe that this is due to the immediate reduction of both hips (within 4 hours) after the patient arrived in the emergency department. In addition, any current disability is actually due to the comminuted talar fracture that had malunited and the patient's noncompliance with physiotherapy.

Closed reduction is preferred unless a fracture is present. Open surgery can be recommended in cases of major fractures or failure of closed reduction. In our patient's case, lack of careful assessment and imaging facilities led to a delay in the diagnosis of the left femoral head fracture.

Finally, the patient underwent a preoperative CT to assess the joint for optimal surgical approach and planning. After the surgery, the patient was restricted from ambulation for 5 weeks, mainly due to the ankle fracture. The patient was also instructed to commence physiotherapy (consisting of passive movement) as soon as possible to alleviate stiffness and muscle wasting, of which he was noncompliant. The patient was fully weight bearing at 8 months after the surgery, with an ankle orthosis.

Despite the odds, 3-month postoperative imaging showed complete healing of the fractures, and after 2 years of consistent follow-up, there were no signs of osteonecrosis.

This is the first reported case of bilateral asymmetric hip dislocation with a rotated Pipkin type II femoral head fracture. Similar case reports in the literature had varying results, one with complete recovery of the hip as in our patient^9^ and others where changes suggestive of osteonecrosis appeared.^10,11^ These cases were associated with acetabular fractures, which is the most common associated fracture in this type of injury.^2^

## Conclusion

Bilateral asymmetric dislocation of the hip is a very rare injury. In our case, both hips were treated with closed reduction, and a delay in diagnosing the left femoral head fracture led to it being operated on 4 days after the accident, using open reduction and internal fixation. Despite this, no signs of osteonecrosis, heterotopic changes, or arthritic changes developed even 2 years after the incident. The prompt reduction of both hips could explain this outcome. Consistent monitoring is necessary to avoid long-term sequelae in case they occur.
